# Malaria Policy Advisory Committee to the WHO: conclusions and recommendations of March 2013 meeting

**DOI:** 10.1186/1475-2875-12-213

**Published:** 2013-06-20

**Affiliations:** 1 Global Malaria Programme, World Health Organization, 20 Avenue Appia 27, Geneva, CH-1211, Switzerland

**Keywords:** WHO, Malaria, Policy making, Treatment efficacy, Fever, Surveillance, Standards, Mosquito control, Malaria vaccines, Resource allocation, Disease elimination, Pregnancy, Prevention

## Abstract

The Malaria Policy Advisory Committee to the World Health Organization met in Geneva, Switzerland from 13 to 15 March, 2013. This article provides a summary of the discussions, conclusions and recommendations from that meeting.

Meeting sessions included: a review of the efficacy of artemisinin-based combination therapy in Guyana and Suriname; the outcomes from a consultation on non-malaria febrile illness; the outcomes from the second meeting of the Evidence Review Group on malaria burden estimation; an update on the review of the WHO Guidelines for the Treatment of Malaria; an update regarding progress on the constitution of the vector control Technical Expert Group; updates on the RTS, S/AS01 vaccine and the malaria vaccine technology roadmap; financing and resource allocation for malaria control; malaria surveillance and the need for a surveillance, monitoring and evaluation Technical Expert Group; criteria and classification related to malaria elimination; the next meeting of the Evidence Review Group on Intermittent Preventive Treatment in pregnancy; an update on the soon-to-be launched Elimination Scenario Planning Tool; and an update on the process for the Global Technical Strategy for Malaria Control and Elimination (2016–2025).

Policy statements, position statements, and guidelines that arise from the MPAC meeting conclusions and recommendations will be formally issued and disseminated to World Health Organization Member States by the World Health Organization Global Malaria Programme.

## Background

The Malaria Policy Advisory Committee (MPAC) to the WHO met from 13 to 15 March 2013 in Geneva, Switzerland, following its meetings in February and September 2012 [1,2]. This article provides a summary of the discussions, conclusions and recommendations from that meeting^a^ as part of the recently established *Malaria Journal* thematic series “WHO global malaria recommendations” [3].

The following sections of this article provide details and references for the background documents presented at the open meeting sessions of the committee on: a review of the efficacy of artemisinin-based combination therapy (ACT) in Guyana and Suriname; the outcomes from a consultation on non-malaria febrile illness; the outcomes from the second meeting of the Evidence Review Group (ERG) on malaria burden estimation; an update on the review of the WHO Guidelines for the Treatment of Malaria; an update from the newly constituted vector control Technical Expert Group (TEG); updates on the RTS,S/AS01 vaccine and the malaria vaccine technology roadmap; financing and resource allocation for malaria control; malaria surveillance and the need for a surveillance, monitoring and evaluation TEG; criteria and classification related to malaria elimination; the next meeting of the ERG on Intermittent Preventive Treatment in pregnancy (IPTp); an update on the soon-to-be launched Elimination Scenario Planning Tool; an update on the process for the Global Technical Strategy for Malaria Control and Elimination (2016–2025).

The MPAC discussion and recommendations related to these topics, which took place partially in closed session, are also included. MPAC decisions are reached by consensus [4]. The next meeting of the MPAC will be 11 to 13 September 2013 [5].

### Report from the WHO global malaria programme

The Director of the WHO Global Malaria Programme (WHO-GMP) updated MPAC members on progress with recommendations from their last meeting [6], in particular highlights from the World Malaria Report (WMR) 2012 [7] and updates from the WHO Regional Offices.

The presentation, on behalf of the Global Malaria Team (WHO-GMP staff and the WHO Regional Malaria Advisors) highlighted the urgent need to improve surveillance systems, which are essential for targeting malaria control at national and subnational levels. Figures showed that the higher the malaria burden in countries, the lower the proportion of cases that are captured by surveillance systems, and the less likely that trends can be reliably assessed. In other words, malaria surveillance systems are weakest where the malaria burden is greatest – the 58 countries in which it is possible to assess trends using data from routine health information systems account for only 15% of the global malaria burden.

Updates were also provided on the global strategic plan for *Plasmodium vivax* control and elimination, due for completion by 2015, and the Malaria Situation Room, a collaborative effort, led by the Roll Back Malaria (RBM) Partnership secretariat and WHO, with support from the African Leaders Malaria Alliance, the International Federation of the Red Cross and Red Crescent Societies, and the Office of the UN Secretary General’s Special Envoy for Malaria and Financing of the Health Millennium Development Goals (MDGs), to track financial flow, commodities, intervention coverage and impact to identify and alleviate bottlenecks. Initially, the Malaria Situation Room will focus on the ten highest burden countries in Africa, as they account for 70% of regional and 56% of the global malaria burden.

In addition, updates were provided on: the soon-to-be-implemented Rapid Access Expansion (RAcE) 2015 programme [8], which will provide support to catalyse the scale-up of integrated community case management of malaria, pneumonia and diarrhoeal disease (iCCM) in five African countries as an integral part of government health services; seasonal malaria chemoprevention (SMC) [9], for which an implementation manual was launched and a training workshop conducted at the end of 2012; the opening of a regional hub in Cambodia as part of the emergency response to artemisinin resistance in the Greater Mekong sub-Region [10], which was endorsed by countries in February 2013 and launched on World Malaria Day; the publication of the third edition of the handbook for the Management of Severe Malaria [11] in early 2013; the publication of four case studies on malaria elimination in October 2012 [12-15], with six more due to be launched in 2013 (Turkey, Philippines, Malaysia, La Reunion, Tunisia, Bhutan) to help national malaria control programmes and other partners contemplating elimination have a better understanding of process involved; and key features of the new funding model at the Global Fund [16].

MPAC commended the work of WHO-GMP and the Regional Offices in helping countries to monitor and reduce their malaria burden. It also highlighted the importance of increasing support to malaria-endemic countries to build human resource capacity to manage malaria programmes, undertake operational research and implement policy recommendations at all levels of the health care system.

### Drug resistance

Therapeutic efficacy monitoring is an essential step in preventing the emergence of artemisinin resistance [17]. When it last met in June 2012 [18], and during its update to MPAC in September 2012 [19], the drug resistance and containment TEG (DRC TEG) recommended that, although there was at the time no evidence of artemisinin resistance outside the Greater Mekong sub-Region, nonetheless surveillance on ACT efficacy outside the sub-Region should continue and be intensified. It encouraged consultation with the DRC TEG by WHO-GMP whenever new data raise concerns.

The DRC TEG and WHO-GMP reported that in early 2013, preliminary results from therapeutic efficacy studies conducted in Suriname and Guyana raised a signal that artemisinin resistance may be emerging in South America in certain areas with a high number of migrants [20,21]. An informal consultation on the emergence of artemisinin resistance in South America, attended by representatives from the Ministries of Health of Suriname and Guyana, the US Centers for Disease Control and Prevention, the US Agency for International Development, WHO, and the chair of the DRC TEG, was held in Washington DC in February 2013 to review the most recent data from Suriname and Guyana.

Given reports of reduced parasite clearance on day three, which is an indication of possible emerging resistance, representatives at this consultation meeting agreed that activities to contain artemisinin resistance, as outlined in the Global Plan for Artemisinin Resistance Containment (GPARC) [17], should be initiated. However, they also agreed that confirmatory studies should be conducted in Suriname and Guyana as soon as possible. This conclusion was fully supported by MPAC, considering that the quality of the microscopy in the Suriname and Guyana studies appeared to be variable. MPAC concluded that it cannot be determined if the signal of possible resistance is real or an artifact resulting from technical problems.

A communication regarding the findings from Suriname and Guyana and short-term actions has been issued by the WHO-PAHO office [22]. If the possibility of the emergence of artemisinin resistance is confirmed by further studies or additional data, various stakeholders, including neighbouring countries, donors and technical partners, will be notified of the data and their implications.

MPAC advised that although many countries in South America have managed to dramatically reduce the number of malaria cases, the findings from Suriname and Guyana highlight the need for all endemic countries to conduct routine monitoring of therapeutic efficacy of anti-malarial drugs. It also recommended that the DRC TEG increase its membership to include a national malaria control programme (NMCP) representative from South America, in addition to two representatives from Southeast Asia.

### Non-malaria febrile illness

Increased diagnostic testing of malaria prior to treatment, paired with decreasing malaria transmission in many areas, has resulted in an increased proportion of febrile patients being diagnosed as not having malaria [23]. However, following the longstanding practice of treating malaria based on the presence of fever alone, health workers may ignore negative test results and still treat the patient with an anti-malarial. This negates the clinical benefits of diagnostic confirmation, wastes valuable anti-malarial drugs, and potentially increases the drug pressure on *Plasmodium* parasites. These problems are exacerbated by the absence of guidance and medicines for the management of non-malaria febrile illnesses.

To help address the lack of guidance, WHO-GMP and the Special Programme for Research and Training in Tropical Diseases (TDR) convened an informal consultation in January 2013 to: (a) review existing evidence and guidance on the management of malaria and non-malaria fevers at primary care and community levels; (b) provide practical recommendations and operational tools based on research findings and successful country experiences for the implementation of integrated management of fevers at peripheral health facility and community level; and, (c) identify and discuss major research gaps [24].

The main conclusions and recommendations from this meeting are:

1. Malaria diagnostic testing and treatment should be deployed as part of programmes promoting the integrated management of fevers.

2. Evidence and lessons learned from implementation studies should be taken into account when scaling-up iCCM.

3. The key elements of the generic iCCM algorithm should not be modified when adopted and implemented in different countries.

4. iCCM programmes should be implemented together with strengthening quality of care in health facilities.

5. Programmes aimed at improving the quality of care of malaria case management in the private sector should also cover the diagnosis and treatment of common non-malaria causes of fever.

6. More studies on aetiologies of fevers need to be undertaken at different levels of health care and in different epidemiological settings, seasons and age groups.

7. Research on new strategies for effective diagnostic testing and treatment of febrile illness should be encouraged, using clinical outcomes as primary study endpoints, in order to modify or expand the diseases which are being targeted by the current WHO algorithms based on health care needs.

The MPAC endorsed the conclusions of the informal consultation and encouraged global malaria control partners to adopt the meeting recommendations as appropriate.

### Malaria burden estimation

The ERG on malaria burden estimation (ERG MBE) met for the second of three planned meetings from 22 to 24 January, 2013 to: (a) review current methods in malaria morbidity and mortality estimation with the participation of the experts involved in the development of currently used methods; (b) achieve consensus on the methods that should be used in the future by WHO; and, (c) identify research that could facilitate the reconciliation of different methodologies and results [25].

As part of its update to MPAC [26], the ERG recommended that for 2013 morbidity estimates, WHO should continue to estimate cases as it does currently, but should vary/test assumptions regarding the value of insecticide-treated net (ITN) effectiveness and investigate malaria positivity among febrile children seeking care *versus* those not seeking care. For 2014 and beyond, in sub-Saharan Africa, it recommended that WHO derive case estimates based on a time-series of *Plasmodium falciparum* parasite rate (*Pf*PR) assembled by the Malaria Atlas Project (MAP) and a refined model of the relationship between prevalence and incidence (including survey data, seasonality information and new covariates). For outside of Africa and in African countries with robust surveillance data, the ERG MBE recommended that morbidity estimates should be based on reported cases as currently undertaken by WHO; as surveillance systems become stronger, more countries will be able to use the Health Management Information System (HMIS).

It also recommended that point estimates and uncertainty ranges should always be presented together, and country consultations should remain integral to the process in order to understand data quality and anomalies and to validate results. The development of a more user-friendly cartographic methodology should also be explored [25].

For malaria mortality estimation, the ERG MBE recommended that for 2013, WHO should estimate malaria deaths as it does currently, but should also derive and apply a case fatality rate for *P*. *vivax* in order to estimate these deaths. It has not yet reached a conclusion on what the recommended approach for mortality estimates should be in 2014 and beyond, as it found that there are substantial limitations in all the current methods. Similar to its recommendations for morbidity estimates, the ERG MBE recommended that point estimates and uncertainty ranges for malaria mortality should always be presented together and country consultations should be an integral part of the estimation process [25].

The ERG MBE had several recommendations to improve the science of malaria burden estimation. For example, for morbidity estimates, it suggested exploring methods of collecting additional prevalence data, e.g., through rapid diagnostic tests (RDTs) at antenatal visits, Expanded Programme on Immunization (EPI) visits, or during school deworming campaigns. For improving mortality estimates, suggestions included novel research to examine age patterns of malaria deaths and the relationship between *Pf*PR and mortality, e.g., case–control studies comparing parasite prevalence in those dying of any cause and controls, and prospective cohort studies of all-cause mortality in relation to malaria exposure. To explore reasons for differing results, the ERG MBE suggested that the Child Health Epidemiology Reference Group (CHERG) should rerun its model using less restrictive verbal autopsy (VA) inclusion criteria, and that the Institute for Health Metrics and Evaluation (IHME) should rerun its model without redistribution of unassigned VA deaths. The ERG MBE asked MPAC to consider the need for a TEG that could provide ongoing guidance to evaluate new estimation methods for both morbidity and mortality as new studies and methods are developed [26]. This was addressed as an agenda item later in the meeting.

The MPAC concluded that while there seems to be a reasonable way forward with respect to the estimation of malaria case numbers, which WHO-GMP will adopt for the next WMR and onwards, the most appropriate method for estimating malaria deaths, particularly among adults for whom VA remains a very crude estimation tool, is unclear. Following an upcoming teleconference, the ERG MBE will decide on the necessity for, and the timing of, its final meeting prior to the next MPAC meeting in September 2013. Several partners, including INDEPTH, expressed a willingness to assist WHO-GMP in continuing to improve malaria burden estimation.

### WHO guidelines for the treatment of malaria

The WHO Guidelines for the Treatment of Malaria (MTGs) provides comprehensive evidence-based guidelines for the formulation of policies and protocols for the treatment of malaria globally; the document was last revised in 2010. The MPAC, at its last meeting, endorsed the plan presented by the Chemotherapy TEG to update the MTGs and for WHO-GMP to publish a third edition. The sub-committee of the Chemotherapy TEG tasked with developing the scope of work for the next edition of the MTGs met in Geneva from 25 to 26 February, 2013 and consensus was reached on the proposed revisions and updates for the third edition of the MTGs [27].

In its update to MPAC [28], the Chemotherapy TEG reported that it would conduct a comprehensive review of existing recommendations in light of any new evidence that might affect each recommendation in its totality, or with regard to the strength of the recommendation. A new section will be included to guide the use of anti-malarials in the prevention of malaria, i.e., intermittent preventive treatment, seasonal malaria chemoprevention, and chemoprophylaxis in travellers. This addition was welcomed by MPAC, which also suggested that it might be useful to receive feedback on the MTGs content from current endusers.

MPAC endorsed the timeline proposed – systematic review completion by the end of 2013 with publication of the revised MTGs in mid-2014 – but added that these were ambitious targets. The major rate-limiting step will be the availability of evidence in a format suitable for systematic review to which the grades of recommendation assessment, development and evaluation (GRADE) methodology, which is the system used by the WHO Guidelines Review Committee, can be applied. The chemotherapy TEG will update MPAC on progress with the systematic reviews at its next meeting in September 2013.

### Malaria vector control

At its last meeting in September 2012, MPAC endorsed the establishment of a vector control TEG (VC TEG) on malaria vector control to review and make recommendations on the use and appropriate mix of malaria vector control interventions for particular situations, including: (a) the adoption of new forms of vector control following recognition of “proof of principle” from the newly established Vector Control Advisory Group (VCAG); (b) the formulation of evidence-based norms, standards and guidelines for the implementation and management of malaria vector control; (c) policy issues related to building capacity for entomological monitoring and optimization of vector control investments; and, (d) identifying gaps in evidence and specific areas of research to improve the management and implementation of malaria vector control.

Following an open call for curriculum vitae (CV) from experts interested in serving on either the VC TEG or the VCAG, a total of 147 applications were received and reviewed by a panel that included external experts. VCAG, focused on vector control tools, including those for other vector-borne diseases such as dengue, will be jointly managed by WHO-GMP and the WHO Neglected Tropical Disease Department (WHO-NTD). The VC TEG, focused on malaria vector control strategies, is managed by WHO-GMP and reports to the MPAC. Members for both groups have now been selected and the groups have been formally constituted [29].

In its update to MPAC [30], the VC TEG, which plans to meet for the first time in July 2013, outlined its work plan, which includes the following outputs, which will be presented for approval at the next MPAC meeting in September 2013: (a) a position statement on methods for maintaining coverage with long-lasting insecticidal nets (LLINs); (b) technical guidance for countries and partners on how to estimate the survival of LLINs from field data on durability; and, (c) technical guidance for countries on how to prioritize malaria vector control interventions when faced with constrained or unstable resources. In addition, the VC TEG plans to draft a technical paper on capacity building for malaria vector control as part of its work plan for 2014.

MPAC welcomed the first three major tasks of the new VC TEG, which will provide urgently needed guidance to countries on malaria vector control, particularly at community level. It also stressed the important role the RBM Vector Control Working Group (VCWG) will play in helping to ensure VC TEG recommendations are implemented. It identified that one type of expertise missing from the VC TEGs membership was social science, and that a social scientist must be included as a core member. WHO-GMP will follow up on this recommendation prior to the first meeting of the VC TEG in July 2013.

### RTS,S/AS01 malaria vaccine

Since its last update to MPAC, the Joint Technical Expert Group (JTEG) on Malaria Vaccines, jointly convened by WHO-GMP and the WHO Department of Immunization, Vaccines and Biologicals (WHO-IVB), met in October 2012 to review the second set of results from the Pivotal Phase 3 trial of RTS,S/AS01, a candidate vaccine developed in partnership between GlaxoSmithKline (GSK) and the PATH Malaria Vaccine Initiative (MVI). These results, since published [31], were also presented in summary to MPAC by MVI [32].

Depending on the timings of regulatory submission, malaria vaccine policy recommendations will be made in late 2015 during a joint session with MPAC and the Strategic Advisory Group of Experts on Immunization (SAGE). These recommendations will be based on all data available up to 2015, including 30 months of follow-up in two different age cohorts, site-specific efficacy data and 12 months’ follow-up of a booster dose given 18 months after the primary series. GSK/MVI have agreed that additional analyses requested by JTEG will be performed prior to 2015, and will form part of WHO’s evidence assessment.

JTEG reported that key policy questions include the duration of protection, whether efficacy varies with transmission intensity, and determining the appropriate age group and schedule for administration [33]. Although the original target group was infants aged six, ten, and 14 weeks, the published results raise the possibility of implementation in children aged five to 17 months. If the protective efficacy is confirmed to be higher in this age group, it would have operational implications, including potentially higher delivery costs. It is too early to draw conclusions about the public health role of RTS,S/AS01. This vaccine will be evaluated as a potential addition to, not a replacement for, integrated approaches of existing preventive, diagnostic and treatment measures tailored to a given endemic setting [34].

Detailed questions and answers regarding RTS,S/AS01 are available on the WHO website [35].

### Malaria vaccine technology roadmap update

MPAC was also updated on the Malaria Vaccine Technology Roadmap [36,37], which was originally launched in 2006 and focused at that time on *P*. *falciparum*, the under-five year old age group, and prevention of severe disease and death. Parts of the 2006 Roadmap are out of date, and it is currently being revised. The updated version includes consideration of both *P*. *falciparum* and *P*. *vivax*. The two new strategic goals include firstly, a focus on clinical disease prevention in endemic areas, and secondly, on transmission reduction that could potentially enable elimination in multiple settings if appropriate vaccines are developed, with a time frame of at least five to ten years for vaccine development. Two sets of WHO preferred product characteristics (PPCs) will be developed in 2013–2014 that will provide technical guidance for vaccine developers at early stages of vaccine research and development to address both of these strategic goals.

MPAC endorsed the concept of WHO PPCs for malaria vaccines, and recommended that MPAC’s input be included as the documents are developed, together with the input of the Strategic Advisory Group of Experts on Immunization (SAGE).

### Financing malaria control

WHO-GMP sought guidance from MPAC on what strategies should be used to allocate limited funds, both globally between countries and internally within countries [38]. Although MPAC advises WHO-GMP on the most effective interventions for malaria control and elimination, current funding levels do not allow for full implementation of these interventions globally. It is important that decisions on resource allocation are based on transparent, clearly defined criteria rather than being driven by political expediency or by those with the loudest voice.

The question of global resource allocation primarily affects international funding for malaria control between countries. In considering equity and health objectives, WHO-GMP presented five hypothetical ways, together with illustrated examples, in which funding for malaria control could be allocated among countries: (a) allocating equal amounts of money per person at risk of malaria; (b) allocating funds in order to provide equal access to interventions; (c) allocating funds according to disease burden, e.g., in proportion to number of deaths or death rates; (d) allocating funds to maximize lives saved; and, (e) allocating funds to equalize health status [39].

For resource allocation within countries, few governments have sufficient resources to achieve universal coverage of all malaria control interventions (vector control, diagnostic testing, treatment, surveillance, management support etc.). As a consequence, they make decisions, in many cases with little guidance, on what blend of interventions should be used, their scale of deployment, and on the populations that should benefit. Of particular relevance are the questions: (a) what interventions should a country invest in if resources are not sufficient to achieve universal coverage of vector control, diagnostic testing and treatment?; and, (b) to which populations should interventions be targeted? Should there be: (i) no targeting, i.e., all populations at risk get an equal share of resources; (ii) targeting to highest transmission areas; or, (iii) targeting to demographically vulnerable groups such as pregnant women and children?

The advice from MPAC for both funders and countries was to always use the guiding public health principle of maximizing health gains to determine how to allocate limited resources between countries and within countries. This principle generally implies that financing and interventions should be targeted to countries and populations with the highest mortality rates, although targeting could be by geographical area or by vulnerable groups, or both. MPAC members felt that these options were not mutually exclusive as long as the guiding principle remained the same.

MPAC accepted that in some cases, global funding politics might interfere with the implementation of this advice; however it did not change what the guiding principle should be, from a technical standpoint. MPAC took great care to stress that maximizing health gains in countries included the principle of continuing investments in places where the disease burden has been reduced through control measures, but where the intrinsic malaria transmission potential remains elevated, in order to avoid malaria resurgence with high mortality and loss of previous gains.

### Malaria surveillance

MPAC considered whether a TEG should be established on surveillance, monitoring and evaluation (SME TEG). The SME TEG would develop guidance on what strategies endemic countries can employ to monitor and evaluate malaria programmes which would include financial tracking, programme coverage, disease trends, and following the advice of the ERG MBE, malaria burden estimation as well [40].

WHO-GMP explained the urgent need for a SME TEG [41]. The past decade has witnessed tremendous expansion in the financing and coverage of malaria control programmes which has led to significant decreases in malaria cases and deaths [7]. However, while there has been much progress in programme implementation, the ability to track programme financing, coverage and impact remains weak, particularly in countries where both burden and malaria control investments are greatest. For example, out of the 99 countries with ongoing malaria transmission, 41 were unable to submit sufficiently complete and consistent data to reliably assess trends in malaria cases. These countries account for 85% of estimated malaria cases [7].

Weaknesses in surveillance, monitoring and evaluation stem partly from the fragmented guidance to countries on how to monitor and evaluate programmes. There has been progress in the development of such guidance in the past decade: WHO-GMP released two surveillance manuals in 2012 [42,43], and the RBM Monitoring and Evaluation Reference Group (MERG) has worked to harmonize household survey indictors for ITN coverage, uptake of IPTp, parasite prevalence and, more recently, diagnostic testing. However, significant gaps remain, such as how to monitor the extent of diagnostic testing and the appropriate use of anti-malarial medicines, which are key components of the *T3*: *Test*. *Treat*. *Track*. initiative [44] launched by the WHO Director General in April 2012.

A principal gap is the lack of updated comprehensive guidance that is specifically useful to NMCP managers and other national and subnational public health staff. RBM MERG has made considerable advances in ensuring that approaches used in large surveys are consistent, but the principal focus has been on deriving information for international monitoring rather than developing guidance to strengthen national surveillance systems.

MPAC endorsed the creation of a SME TEG, noting that guidance to countries should be consistent with WHO recommendations, and there should be no confusion on what the indicators for monitoring programme coverage should be. Such guidance should be reviewed on a regular basis, in conjunction with latest MPAC recommendations or methodological developments, in order to reflect current best practice. WHO-GMP should work closely with RBM and its working groups to help ensure that the guidance from the SME TEG is implemented at country level.

WHO-GMP will begin a call for CV of interested experts, and report on progress in constituting the SME TEG at the next MPAC meeting in September 2013. It will also enable a natural handover from the time-limited ERG on malaria burden estimation to the new TEG. WHO-GMP will work closely with RBM to ensure that the SME TEG and the MERG complement and coordinate with each other.

### Criteria and classification related to malaria elimination

The purpose of this session was to introduce possible development of a definition of and criteria for malaria elimination at the subnational level [45]; these will be presented for decision at a future MPAC meeting.

While there is mention of the concept of subnational malaria elimination in some WHO documents, it has been suggested by WHO Member States and their implementing partners that there is a need for formal WHO guidance to countries regarding the process of achieving, maintaining and documenting subnational elimination. Experience from the Philippines suggests that: (a) such national processes should emulate WHO certification; (b) a clear distinction should be made between the roles of the national authorities and those of the subnational areas under consideration for malaria-free status; and, (c) emphasis should be placed on the capacity of the subnational administrative area to achieve and maintain malaria-free status with limited central financial and technical support. However, this might need to be applied with flexibility in the case of, for example, small island provinces.

MPAC concluded that there is a need for WHO guidance to countries about handling subnational elimination, for example at the state and province levels in countries such as India and China, and requested WHO-GMP to present a clear proposal at a future meeting. Subnational elimination targets, should countries choose to pursue them, could be important internal milestones for countries, as well as being potentially important international milestones, especially in larger countries. It was suggested that at a future meeting, MPAC should review current criteria for WHO certification and discuss the possible need for a procedure for decertification.

### Intermittent preventive treatment in pregnancy

WHO-GMP provided MPAC with a brief update on progress with its IPTp recommendations [46]. In October 2012, following MPAC recommendations to update the IPTp-sulphadoxine-pyrimethamine (IPTp-SP) policy to provide SP at each scheduled antenatal care visit [47], WHO published the recommendation and urged national health authorities to disseminate it widely and to ensure its correct application. Based on initial feedback from national programmes and implementing partners, WHO-GMP and the WHO Reproductive Health and Research Department (WHO-RHR) developed a policy briefing paper to offer additional background information, more explanations on operational aspects, a compilation of the scientific evidence, and a set of frequently asked questions on IPTp-SP [48].

WHO-GMP reported that new evidence will be available for review by the ERG IPTp in July 2013, including results from: (a) a series of IPTp-SP studies by the Malaria in Pregnancy Consortium (MIPc) and the US President’s Malaria Initiative (PMI) evaluating the association between SP resistance and IPTp-SP effectiveness; and, (b) two randomized clinical trials on the efficacy and safety of mefloquine for IPTp, in the context of ITNs.

In addition, simplified protocols are being developed to monitor the potential impact of SP resistance on IPTp-SP effectiveness and to monitor the programmatic determinants of IPTp-SP effectiveness.

The ERG IPTp will reconvene in July 2013 to: (a) review the evidence regarding the contribution of SP resistance to IPTp effectiveness; (b) finalize the core protocol to monitor the impact of SP resistance on IPTp-SP effectiveness; (c) review evidence on the efficacy and safety of mefloquine for IPTp compared to SP (for all women) and to daily co-trimoxazole prophylaxis (for HIV + pregnant women); and, (d) develop draft policy recommendations on the contribution of SP resistance to IPTp effectiveness and monitoring methods, as well as on the efficacy and safety of mefloquine for IPTp for consideration by the MPAC in September 2013.

Because of the safety concerns related to the use of mefloquine, MPAC urged WHO-GMP to undertake a safety review of this drug with particular reference to its neuropsychiatric side-effects, which are also relevant for the recommendations for its use for chemoprophylaxis in the next (third) edition of the WHO Guidelines for the Treatment of Malaria [49].

### Elimination scenario planning toolkit

An Elimination Scenario Planning (ESP) toolkit is currently in the process of being finalized after being field tested using data from The Gambia and Senegal in 2012, and will be released online, with an accompanying manual, in the next few months. WHO-GMP updated the MPAC on the ESP toolkit, and requested advice on potential new directions following its initial release [50,51].

The ESP toolkit, developed by WHO-GMP with partners from the Clinton Health Access Initiative (CHAI), Imperial College London, and the Global Health Group at the University of California San Francisco (GHG/UCSF), covers the technical, operational and financial aspects of malaria elimination, and provides realistic timelines for programmes moving from the control to the elimination phase of malaria programme operations. The toolkit includes a manual that reviews elimination concepts and guides users through the feasibility of malaria elimination. The manual is linked to software that models malaria transmission, currently limited to *P*. *falciparum* in Africa, which allows users to explore the effect of a range of combinations of malaria control interventions in order to achieve elimination. Feedback from field testing by malaria control programme staff and researchers from The Gambia and Senegal has been positive.

During the development of the toolkit, WHO-GMP and partners recognized that a similar approach could be used for malaria programme planning in other settings. WHO-GMP sought advice from MPAC on: (a) whether the ESP toolkit should be modified to function as a general programme planning tool; (b) whether it should be extended to address scenarios of low transmission *P*. *falciparum* outside of Africa; and, (c) whether it should be extended to cover settings where *P*. *vivax* is predominant.

Although at present the toolkit is focused on planning for potential elimination scenarios, many of the concepts covered in the manual regarding technical and operational aspects of implementing interventions, including the transmission software itself, are applicable to countries that have near-term goals other than elimination. With a steadily growing list of intervention tools, one aspect of the software that countries in the control programme phase may find useful is the ability to explore combinations of interventions, and their projected cost. MPAC members welcomed this development, and were broadly supportive of extending the toolkit, particularly for *P*. *falciparum* outside Africa, but felt that this should not be rushed. For example, extending the toolkit for *P*. *vivax* settings would be in line with the current work of WHO-GMP in the development of a *P*. *vivax* strategy, and could be timed for release at the same time.

In general, MPAC members welcomed the development of the ESP toolkit that NMCPs could use to add rigour to programme goal setting and policy development, as well as in planning and budgeting for interventions. This view was supported by the NMCP managers, and WHO Regional Malaria Advisors present at the meeting.

### Global technical strategy (2016–2025)

WHO-GMP provided an update to MPAC on progress since its last meeting where members called for an overarching review of the malaria strategy mix to underpin planned revisions to the Global Malaria Action Plan (GMAP) [52]. After a brief historical perspective – the last Global Strategy for malaria was launched in 1993 and was a highly time- and resource-intensive process – WHO-GMP outlined some of the key issues it is grappling with as it moves forward with the Global Technical Strategy 2016–2025 (GTS) [53].

One of these is timing, since a policy recommendation on the RTS,S vaccine will not be made until late 2015, at the earliest. Although seeking formal World Health Assembly (WHA) endorsement of the GTS will increase the engagement of Member States and elevate the political profile of the strategy, it will also have implications on the timing for developing, finalizing and launching the GTS. The process for country and regional consultation in developing the GTS was discussed. Broad endemic country input is critical. However, it is not feasible or efficient to replicate the lengthy and costly process used to develop the last global strategy. Moreover, there are already many region-specific strategies. WHO-GMP also highlighted the importance of working closely with RBM to harmonize the development of the GTS and the update of the GMAP.

The MPAC provided guidance on how to address these issues. It felt strongly that the timing for developing the GTS should not be bound by the anticipated recommendation for the RTS,S vaccine in 2015. There will be multiple new tools on the horizon; their development should be included, but guidance will need to be provided as the evidence emerges for any new tools or approaches. Members envisioned that the GTS should be a living document – a clear and concise technical strategy that can be updated as necessary and adapted for regional and national use to produce more detailed implementation plans that are relevant to the local context. This is one reason why it will be extremely important to engage regional and national experts in developing the GTS. Suggestions based on members’ experience with other global strategies, specifically the Global Vaccine Action Plan, included using a combination of web consultations and add-ons to already scheduled regional and national meetings in order to seek input.

MPAC recommended that the GTS be put forward to the sixty eighth session of the WHA in 2015 for endorsement. It also suggested that the GTS and GMAP be developed in a collaborative process, and be launched together as companion documents in the second half of 2015. One possible way that might help this process was to have some overlap in the GTS and GMAP Steering Committees.

MPAC advised that WHO-GMP establish an internal working group to help lead the process under the advice of MPAC and a Steering Committee. This working group will develop an initial draft of the GTS prior to seeking broader consultation from regions and counties.

## Discussion

The wording for recommendations was finalized by MPAC during their closed session following the two-and-a-half days of open sessions; conclusions have been included in the summaries of the meeting sessions above, and links to the full set of meeting documents have been provided as references.

Position statements and policy recommendations made by the MPAC are approved by the WHO Director-General, and will be formally issued and disseminated to WHO Member States by WHO-GMP or if more appropriate, the WHO Regional Offices. Conclusions and recommendations from MPAC meetings are published in the *Malaria Journal* as part of this series.

MPAC provided suggestions for the agenda for its next meeting to the WHO-GMP Secretariat. Feedback will also be given to and received from the global malaria community at the RBM Board meeting in May 2013, and through the publication of and correspondence regarding this article.

Ongoing engagement with and attendance by interested stakeholders at MPAC meetings continues to be encouraged. In addition to open registration for MPAC meetings, which will continue (via the WHO-GMP website starting in July) and attendance by four standing observers (RBM, the Global Fund, UNICEF, Office of the UN Special Envoy for malaria), the active participation of seven rotating NMCP representatives, and all six WHO Regional Malaria Advisors, was strongly welcomed.

## Conclusion

The meeting feedback received from participants and observers [54], and MPAC members themselves, was very positive. Having met three times to date, the format of MPAC meetings and its feedback loops with other advisory bodies and stakeholders is beginning to settle, although it remains an evolving process. WHO-GMP and the MPAC continue to strongly welcome feedback, support and suggestions for improvement to MPAC meetings from the global malaria community.

The next meeting of the MPAC will take place from 11 to 13 September 2013 in Geneva, Switzerland. Further information, including the agenda and details on how to register, will be made available in July 2013 on the MPAC page of the WHO-GMP website, although questions are welcome at any time [5].

## Endnotes

^a^ The complete set of all MPAC March 2013 meeting-related documents including background papers, presentations, and member declarations of interest can be found online at http://www.who.int/malaria/mpac/mar2013/en/index.html.

## Abbreviations

MPAC: Malaria Policy Advisory Committee; ACT: Artemisinin-based combination therapy; ERG: Evidence review group; TEG: Technical expert group; IPTp: Intermittent preventive treatment in pregnancy; WHO-GMP: WHO Global Malaria Programme; WMR: World malaria report; RBM: Roll Back Malaria Partnership; MDGs: Millennium development goals; iCCM: Integrated community case management; SMC: Seasonal malaria chemoprevention; DRC TEG: Drug resistance and containment TEG; GPARC: Global plan for artemisinin resistance containment; NMCP: National malaria control programme; ERG MBE: ERG on malaria burden estimation; ITN: Insecticide-treated net; PfPR: *P*. *falciparum* parasite rate; HMIS: Health management information system; RDT: Rapid diagnostic test; EPI: Expanded programme on immunization; CHERG: Child health epidemiology reference Group; VA: Verbal autopsy; IHME: Institute for Health Metrics and Evaluation; MTGs: WHO guidelines for the treatment of malaria; GRADE: Grades of recommendation assessment development and evaluation; VC TEG: Vector control TEG; VCAG: Vector control advisory group; CV: Curriculum vitae; WHO-NTD: WHO Neglected Tropical Disease Department; LLIN: Long-lasting insecticidal nets; VCWG: RBM vector control working group; JTEG: Joint technical expert group; WHO-IVB: WHO Immunization Vaccines and Biologicals Department; SAGE: Strategic advisory group of experts; PPCs: WHO preferred product characteristics; SME TEG: Surveillance monitoring and evaluation TEG; MERG: RBM monitoring and evaluation reference group; IPTp-SP: Intermittent preventive treatment of malaria in pregnancy using sulphadoxine-pyrimethamine; WHO-RHR: WHO Reproductive Health and Research Department; ESP: Elimination scenario planning; GMAP: Global malaria action plan; GTS: Global technical strategy 2016–2025; WHA: World Health Assembly.

## Competing interests

The authors declare that they have no competing interests.

## Authors’ contributions

All authors listed below have equally contributed to the article. All authors have read and approved the final version of the manuscript.

## Authors’ information

WHO Malaria Policy Advisory Committee Members

• Salim Abdulla, Ifakara Health Institute, Dar Es Salaam, United Republic of Tanzania

• Pedro Alonso, Centre for International Health and Research, Barcelona, Spain

• Fred Binka, University of Ghana, Accra, Ghana

• Patricia Graves, James Cook University, Cairns, Australia

• Brian Greenwood, London School of Hygiene and Tropical Medicine, London, UK

• Rose Leke, University of Yaoundé, Yaoundé, Cameroon

• Elfatih Malik, Ministry of Health, Gezira, Sudan

• Kevin Marsh, Kenya Medical Research Institute, Kilifi, Kenya

• Sylvia Meek, Malaria Consortium, London, UK

• Kamini Mendis, Colombo, Sri Lanka

• Allan Schapira, Legazpi City, Philippines

• Laurence Slutsker, Centers for Disease Control and Prevention, Atlanta, USA

• Marcel Tanner, Swiss Tropical Public Health Institute, Basel, Switzerland

• Neena Valecha, National Institute of Malaria Research, New Delhi, India

• Nicholas White, Mahidol University, Bangkok, Thailand

WHO Malaria Policy Advisory Committee Secretariat

• Andrea Bosman, WHO Global Malaria Programme, Geneva, Switzerland

• Richard Cibulskis, WHO Global Malaria Programme, Geneva, Switzerland

• Valerie D’Acremont, WHO Global Malaria Programme, Geneva, Switzerland and Swiss Tropical and Public Health Institute, Basel Switzerland

• Bianca D'Souza, WHO Global Malaria Programme, Geneva, Switzerland and London School of Hygiene and Tropical Medicine, London, UK

• Michael Lynch, WHO Global Malaria Programme, Geneva, Switzerland

• Abraham Mnzava, WHO Global Malaria Programme, Geneva, Switzerland

• Vasee Moorthy, WHO Immunization, Vaccines and Biologicals Department, Geneva, Switzerland

• Robert Newman, WHO Global Malaria Programme, Geneva, Switzerland

• Peter Olumese, WHO Global Malaria Programme, Geneva, Switzerland

• Aafje Rietveld, WHO Global Malaria Programme, Geneva, Switzerland

• Pascal Ringwald, WHO Global Malaria Programme, Geneva, Switzerland
